# Adapting WHO drowning prevention strategies for children in Indonesia: barriers, enablers, and policy perspectives for LMICs

**DOI:** 10.1016/j.lansea.2025.100697

**Published:** 2025-12-02

**Authors:** Muthia Cenderadewi, Richard C. Franklin, Prima B. Fathana, Susan G. Devine

**Affiliations:** a Discipline of Public Health and Tropical Medicine, CMD, James Cook University, Townsville, Queensland, 4811, Australia; b Faculty of Medicine, University of Mataram, West Nusa Tenggara, 83127, Indonesia; c Royal Life Saving Society – Australia, Sydney, New South Wales, 2007, Australia

**Keywords:** Drowning, Health promotion, Health policy, Social determinants of health, Low- and middle-income countries

## Abstract

Drowning is among the top five causes of child mortality in some parts of Indonesia yet remains neglected in national health policy. This study explored how World Health Organization (WHO)-recommended drowning prevention strategies can be adapted in Indonesia through seven focus group discussions with 62 parents of children under five and community leaders across coastal and inland villages in West Nusa Tenggara Province. Participants identified barriers including financial constraints, rural inaccessibility, and sociocultural norms, alongside enablers such as community support for swimming lessons, supervised childcare, and first-aid training. Findings highlight the need for affordable and culturally appropriate interventions. Policy priorities include integrating swimming lessons into school curricula, subsidising community-based swimming programs, establishing supervised childcare centres, expanding first-aid training for community members as bystanders, and strengthening cross-sector coordination.

## Introduction

Drowning is a leading but under-recognised cause of child mortality in low-income and middle-income countries (LMICs).^1^, ^2^, ^3^, ^4^, ^5^ Despite ranking among the top five causes of death in children under five, it remains absent from Indonesia's child health policy agenda.^1^^,^^4^, ^5^, ^6^ Unlike other child survival priorities such as diarrhoea, pneumonia, or malnutrition, drowning has received limited policy attention, leaving major gaps in prevention and preparedness in Indonesia.^6^ This neglect undermines progress towards the Sustainable Development Goals (SDGs), particularly SDG 3.2 on ending preventable child deaths, which includes deaths from drowning.^7^^,^^8^

Indonesia, with more than 17,500 islands and frequent hydrometeorological disasters, faces an especially high risk of drowning.^9^ Despite a decline in national rates from 2005 to 2019, mortality among children under five remains alarmingly high at 9.7 per 100,000, nearly double the global average and several times higher than in high-income countries such as Australia and the United States.^10^, ^11^, ^12^ Risks are also not evenly distributed: provinces such as Papua, Kalimantan, Sulawesi, Maluku, and Nusa Tenggara consistently report higher burdens, reflecting geographic disparities and underscoring the need for locally adapted prevention strategies.^13^ However, the understanding of effective measures in Indonesia remains limited.^6^

Indonesia's decentralised governance structure adds further complexity. While national ministries provide strategic direction, provinces, districts, and villages hold responsibility for planning and implementation. This arrangement allows adaptation to local contexts but also risks fragmentation, weak coordination, and uneven coverage across regions and sectors, particularly for cross-cutting issues such as injury prevention. These governance dynamics create significant challenges for integrating drowning prevention into existing child health, education, and disaster risk reduction programmes, often resulting in policy gaps and inconsistent implementation across the country.^14^^,^^15^

The World Health Organization (WHO) recommends a package of evidence-based interventions, including supervised safe spaces, physical barriers, swimming lessons for school-age children, disaster preparedness, and first-aid training for bystanders, supported by cross-sectoral governance and communication strategies.^1^^,^^2^ In Indonesia, however, no national strategy specifically addresses drowning prevention.^7^ Existing child health and injury prevention policies also give little or no attention to drowning, despite its ranking as a leading cause of under-five mortality. Responsibility for relevant measures is scattered across ministries, including health, education, social affairs, disaster management, and transport, with limited coordination and no clear national framework. Moreover, little evidence is available on the feasibility, cultural acceptability, and sustainability of these interventions within Indonesia's diverse local contexts, leaving drowning prevention marginalised in both policy and practice.^7^

Therefore, this paper draws on community perspectives from West Nusa Tenggara Province to examine how WHO drowning prevention recommendations could be operationalised within Indonesia's policy architecture. By identifying locally grounded barriers and enablers, we outline policy options that can accelerate progress toward Indonesia's child survival commitments under the SDGs and generate lessons for other LMICs facing similar governance and resource challenges.

## Methods

This study used qualitative focus group discussions with community members across both coastal and inland areas of West Nusa Tenggara Province of Indonesia. This study is a part of a broader mixed-methods inquiry into unintentional drowning in Indonesia, which included a scoping review^6^ and a population-based retrospective cohort study.^13^ Employing an exploratory qualitative approach, the study was designed to further explain high drowning mortality rates among children under-five in eastern Indonesia including in West Nusa Tenggara (WNT) Province,^13^ by exploring local perceptions of child drowning risks, barriers to water safety measures, and recommendations for improving prevention efforts. For transparency, while keeping the article focused on health policy perspectives, detailed methods including study design, sampling, moderator guide's questions and probes, stages of thematic analysis, and researcher reflexivity on positionality are reported in full elsewhere.^16^^,^^17^

The research was conducted across seven villages in all districts (West Lombok, North Lombok, East Lombok, Central Lombok, and Mataram) of Lombok Island, WNT Province, covering diverse coastal and inland areas to explore drowning incidents in various settings. WNT was selected as study sites due to its high under-five drowning mortality rates (12.6/100,000 for males and 6.1/100,000 for females in 2019)^13^ and high all-cause under-five mortality rate (29/1000 live births in 2022),^18^ both being one of the highest nationwide. The province's predominantly rural nature, high population density, and significant economic inequalities, with over 19% of its 5 million residents living below the national poverty threshold of less than USD 0.7 per day, also influenced the selection.^19^ Lombok is culturally diverse and predominantly Sasak speaking, with Indonesian used as the formal language.^19^

Indonesia operates under a decentralised governance system with authority distributed across national, provincial, district/city, subdistrict, and village levels. At the national level, ministries and coordinating agencies are responsible for setting policy direction, regulations, and national funding frameworks. These policies are operationalised by subnational governments at successive levels of administration: 38 provinces led by governors; over 500 regencies (‘*kabupaten’*) and cities (‘*kota’*) administered by regents (‘*bupati’*) and mayors (‘*walikota’*); and more than 83,000 villages (‘*desa’*) and their urban counterparts (wards, or ‘*kelurahan’*), each governed by elected or appointed heads. This administrative structure enables policy implementation to be tailored to diverse local needs.^14^^,^^15^

Participants were eligible if they were parents of children under five or community leaders in densely populated coastal or inland water areas of Lombok Island, WNT.^16^ Recruited community leaders included village chiefs, elders, religious figures, and community health workers, most of whom were also parents or grandparents of children under-five.^16^ This demographic composition ensured a clear understanding of the community context surrounding child drowning. Participants were recruited using purposive sampling to ensure focused representation across a wide geographic spread of Lombok based on the inclusion criteria. Furthermore, snowball sampling was also employed to provide access to individuals who may not be easily identifiable through conventional sampling.^16^ This composition ensured the study captured both household-level and leadership perspectives on child drowning and community prevention.

Seven focus group discussions (n = 62) were held at various community locations from October 2023 to March 2024, until data saturation was achieved. The moderator guide, informed by the Health Belief Model (HBM)^20^ and a previous scoping review,^6^ was pilot tested with a small sample of parents in the study area, as outlined elsewhere.^16^ Feedback from research team members and participants on the guide's relevance and clarity led to minor adjustments. Focus group discussions, each lasting 50–60 min, were led by the lead Indonesian researcher proficient in Indonesian and local Sasak language, supported by a note-taker. All discussions were audio-recorded, transcribed, and translated into English.^16^

**Fig. 1:**
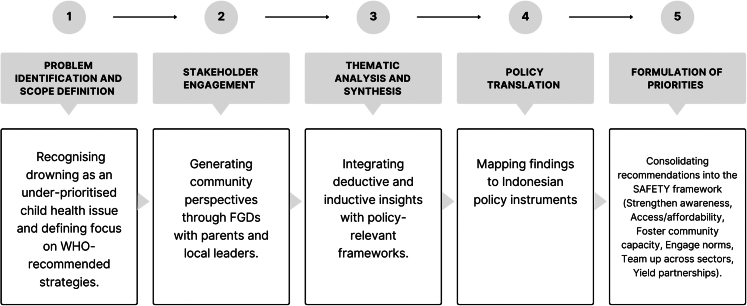
Phases of the research priority-setting process.

NVivo software (version 20) supported data management and analysis. The detailed analytic procedure is reported elsewhere,^16^ with illustrative verbatim transcripts provided in the Appendix (Supplementary Table S1), while the main text presents thematic syntheses with interpretive emphasis on policy implications. Beyond the thematic analysis, to provide a clear account of how community perspectives were translated into policy priorities, the study also followed a structured five-phase process (Fig. 1).

## Results

Sixty-two participants, including parents of children under five and village community leaders, joined the focus groups. The majority were female (75.8%, n = 47), aged between 25 and 44 (51.6%, n = 32) and did not complete primary education (58.1%, n = 36). Most were mothers of children under five (53.2%, n = 33), followed by village community leaders (37.1%, n = 23) and fathers of children under five (9.7%, n = 6) (Table 1).^16^

**Table 1:** Focus group discussion (FGD) participants by group, type, sex, age range, and education level (n = 62).

FGD	Participants composition	M/F	Age range	Education level	Total
1	Mothers (5), Fathers (1), Village Community Leaders (5)	4/7	18–55+	Mostly did not complete primary education (11)	11
2	Mothers (6), Fathers (3), Village Community Leaders (3)	4/8	25–54	Mostly did not complete primary education (10)	12
3	Mothers (8), Village Community Leaders (4)	2/10	25–55+	Half completed primary education (6), half did not (6)	12
4	Mothers (3), Village Community Leaders (3)	0/6	25–55+	Mostly completed primary education (4)	6
5	Mothers (5), Village Community Leaders (5)	3/7	25–55+	Half completed primary education (6), some completed high school (4)	10
6	Mothers (3), Father (1), Village Community Leaders (1)	1/4	35–55+	Most completed high school (3)	5
7	Mothers (3), Father (1), Village Community Leaders (2)	1/5	25–55+	Some did not complete primary education (2), some completed primary (2), some completed high school (2)	6
**Total**	**Mothers (n** = **33; 53.2%), Fathers (n** = **6; 9.7%), Leaders (n** = **23; 37.1%)**	**15 (24.2%)/47 (75.8%)**	**18**–**55+**	**Did not complete primary education (n** = **36; 58.1%), completed primary education (n** = **14; 22.6%), completed high school (n** = **12; 11.6%)**	**62**

Four main themes emerged during the data analysis: a) Perceptions of acceptability towards WHO-recommended interventions; b) Barriers of drowning intervention implementation; c) Sociocultural considerations of implementing interventions; d) Enablers that facilitate participation in interventions. Representative verbatim excerpts are provided in Appendix (Supplementary Table S1).

### Perceptions of acceptability towards WHO-recommended interventions

Across groups, participants expressed broad endorsement of all six WHO-recommended strategies. This general support reflects both an awareness of drowning risks and a recognition that preventive measures are urgently needed, even if communities lacked prior exposure to structured interventions.

Swimming lessons were viewed as the most tangible and directly beneficial intervention. Parents noted that school-based programs existed sporadically, but these were infrequent (once or twice a year), irregularly accessible, and often limited to secondary schools. Parents emphasised that such limited exposure was insufficient to ensure children's survival skills, especially for younger children at higher risk. Several participants highlighted the prohibitive cost and distance of accessing private lessons. Fathers also noted practical challenges, such as the need to accompany children due to restrictions on their mobility (e.g., not being able to ride motorbikes). This demonstrates both the perceived value of swimming skills and the structural barriers to access. Consequently, parents would like to see government-supported community swimming programs, delivered by locally trained instructors, to enhance children's swimming skills and encourage broader participation.

Supervised childcare centres were widely supported, particularly among mothers who felt overburdened with household responsibilities. Participants supported establishing supervised safe places such as childcare and early childcare education centres in their community to prevent children from playing unsafely near rivers, irrigation ditches, or wells. However, while they emphasised supervising school-aged children in these centres, there was less emphasis on the need to supervise preschool-aged children. In addition, while the idea of community-run centres was positively received, several participants indicated that sustainability would depend on external support, particularly from government, to provide training, infrastructure, and financial subsidies for the salaries of participating community members.

First aid training for parents and community members was another priority. Across both male and female groups, participants admitted lacking knowledge of resuscitation or safe rescue techniques. While some had seen resuscitation demonstrations on television, few felt confident to act in an actual drowning emergency. Mothers especially requested practical, hands-on sessions with mannequins or equipment to build confidence, as they were predominantly regarded as the primary caregivers responsible for children's safety at home. This highlights both awareness of the need and readiness to engage if resources are provided.

Infrastructure-related measures, such as covering wells or fencing streams, were welcomed when feasible. Many participants shared experiences of children falling into open wells or unsupervised streams, hence many community members have taken proactive measures to cover their wells as a safety precaution and underscored the importance of physical barriers. However, there was recognition that such measures must not interfere with daily water use, making local adaptation essential. In addition, there was no mention of installing door barriers or playpens to create safe play areas at home or childcare centres, underscoring limited knowledge or cultural familiarity with playpens and their potential as practical, affordable options for physical barriers.

Flood and tsunami preparedness was raised as particularly relevant in coastal villages. Participants described wanting clearer communication from government agencies during emergencies, including timely warnings and guidance.

Enforcement of maritime safety regulations was perceived as almost entirely absent, with participants noted that life jackets were rarely available or enforced on ferries and small boats, despite frequent travel between islands. Parents considered this a major gap in regulation.

Collectively, these findings indicate high acceptability of WHO strategies but highlight an expectation that implementation will require government leadership, investment, and adaptation to community realities. Policy frameworks should therefore create enabling conditions, such as training local instructors, allocating provincial/district/village funds, and embedding water safety into school curricula as well as community health outreach.

### Barriers of drowning intervention implementation

While acceptability was high, participants identified a range of barriers that could undermine implementation and sustainability.

Limited awareness of drowning risks was a recurrent theme. Most participants admitted that drowning was not seen as a major community health issue compared to diarrhoea, fever, or malnutrition. This lack of prioritisation was especially evident in inland areas, where proximity to open water was less constant than in coastal communities. Such perceptions appeared to limit prioritisation of drowning relative to other health concerns, particularly when families face competing health and financial concerns.

Financial constraints were consistently described as the most significant barrier. Parents emphasised the inability to afford transport to swimming pools, entry fees, or additional expenses such as pocket money for children. The distance to swimming and childcare facilities also added financial strain for many parents in rural communities, especially those without vehicles and with limited access to public transportation. Fathers and mothers alike explained that household budgets were already stretched, and paying for lessons more than once or twice a year was unrealistic. To address these barriers, participants identified schools as a valuable entry point for implementing swimming lessons, citing the potential to reduce costs by integrating them into the formal education system. The financial barrier also extended to childcare centres, where parents anticipated that even minimal fees would deter attendance.

Geographic barriers compounded financial challenges. In rural and coastal areas, the nearest swimming pools were often several kilometres away, requiring costly transport. Mothers in particular highlighted difficulties in accompanying children over long distances, especially when they also had younger children at home. Participants emphasised that interventions delivered far from communities would be ineffective, regardless of quality.

Reluctance to delegate child supervision emerged as another barrier. Parents expressed strong cultural and emotional reservations about entrusting their children to non-family members, even within structured childcare centres. While many supported the concept, there was concern that parents would not fully use such centres due to a lack of trust. This barrier was especially pronounced among mothers, who worried about safety and moral guidance when children were under the supervision of outsiders. In addition, some participants doubted community members' ability to engage and participate as caretakers and organisers in community-based childcare centres due to their own domestic and caregiving duties, possibly affecting the program's long-term sustainability.

Physical constraints were also identified. Communities often lacked suitable land or buildings to host childcare centres, with participants noting that limited space, even within homes, posed a barrier to establishing such facilities. In addition, open wells and ditches, essential for daily water use and flood management, could not always be covered without creating new risks. These challenges highlight the tension between theoretically sound solutions and the practical realities of low-resource settings.

Together, these findings indicate that financial, geographic, sociocultural, and physical barriers are likely to shape the feasibility of drowning prevention interventions. For policy and program design, this underscores the importance of approaches that are sensitive to cost, accessibility, and cultural expectations if interventions are to be sustainable in resource-limited settings.

### Sociocultural considerations of implementing interventions

Participants’ perspectives underlined how sociocultural norms were shaped, and influenced how interventions were viewed, as well as highlighting the importance of cultural tailoring.

Gender norms influenced parental comfort with swimming lessons. Parents of adolescent girls were particularly concerned about male instructors and preferred female-led sessions. Fathers and mothers both stressed the need for parental presence at lessons to ensure safety and propriety. Swimwear was another sensitive issue: some parents feared that tight swimsuits would be culturally unacceptable, though others pointed out that modest alternatives now exist. These perspectives indicate that policy and program design may require accommodation of gender norms to ensure inclusivity.

Parental supervision norms were also influential. Parents across groups articulated that they would not feel comfortable leaving children unsupervised during lessons, even with trained instructors. Observing lessons was seen not just as a safeguard but also as a parental responsibility. This cultural expectation may require program designs that integrate parental presence into lesson delivery.

The age for swimming lessons was contested. Some parents, particularly younger mothers, favoured enrolment from around five years of age, citing early skill acquisition as beneficial. Others, particularly older parents and community leaders, leaned towards adolescence, citing safety concerns and fears of younger children being overwhelmed. These differences suggest that uptake may vary by household norms, requiring flexible age-appropriate approaches.

Overall, these sociocultural findings indicate that acceptability of drowning prevention interventions is shaped by gender, supervision, and age norms. Flexibility, sensitivity to local expectations, and opportunities for adaptation at the community level will be crucial for successful implementation.

### Enablers that facilitate participation in interventions

Despite barriers, participants identified several enablers that could promote adoption and sustainability of WHO-recommended drowning interventions in their community.

Government and multisectoral commitment was seen as essential. Agencies such as the Indonesian National Search and Rescue Agency (BASARNAS), the Child Protection Agency, and the Department of Tourism were mentioned as potential leaders in raising awareness and delivering training, with local government highlighted as primarily responsible for child drowning prevention. However, participants did not always identify the agencies with formal mandates for drowning prevention, such as the Ministry of Health, Ministry of Education, or Disaster Risk Reduction authorities, reflecting limited familiarity with relevant policy actors. Notably, there was no mention of non-governmental stakeholders.

Mass media and social media were highlighted as powerful tools to increase community awareness. Parents explained that reports of drowning incidents, particularly involving celebrities or widely shared tragedies, made them more vigilant. This suggests that well-designed media campaigns could shift perceptions of drowning risk and encourage participation.

Financial subsidies were another key enabler. Parents explained that providing free or subsidised swimming lessons and access to childcare centres, as well as stipends for volunteer supervisors in community-driven childcare centres, were viewed as essential. Mothers particularly emphasised that even small financial incentives could motivate community members to participate in the community childcare centres, while subsidies would remove the main obstacle to participation.

Collectivist traditions in caregiving were identified as strengths. Participants noted that neighbours and extended family commonly help care for children when parents are busy. This cultural norm could be leveraged to support community-run childcare centres, provided adequate training and oversight were in place.

Together, these findings point to several practical levers: government support, financial assistance, media mobilisation, and collectivist norms, that communities believe would make drowning prevention strategies more acceptable and sustainable. Although participants did not articulate specific financing mechanisms or legislative pathways, their perspectives suggest potential entry points for aligning local practices with existing national frameworks.

## Discussion

This study confirms strong community support for WHO-recommended drowning prevention strategies, but their implementation in Indonesia is shaped by embedded socioeconomic and cultural factors. Participants emphasised the need for affordable, culturally appropriate, and locally accessible programs, especially swimming lessons, safe play areas, and supervised childcare (Supplementary Table S1). While aligned with WHO guidance, these preferences also reveal barriers in awareness, infrastructure, and institutional support. Participants underlined that the success of such interventions also depends on supportive intersectoral coordination and resourcing which are a critical determinant of whether such interventions can be sustainably implemented (Supplementary Table S1). This underscores the need for sustainable, context-sensitive, low-cost solutions that are backed by both community initiative and structural support across all government levels.

However, this study highlights meaningful community engagement in co-design and implementation depends on first equipping communities with foundational knowledge of drowning risks and prevention strategies. Early investment in education and awareness is therefore essential to support sustainable, locally driven solutions.

### Recognising community capacity and constraints

This study involved participants from rural and peri-urban communities with limited formal education and minimal policy exposure. While they did not articulate technical solutions, they contributed valuable, experience-based insights into what works, and what does not, at the community level. On the community capacity side, participants proposed low-cost, pragmatic strategies rooted in local realities:

• Community-based swimming lessons led by local instructors to reduce access and cost barriers.
• Informal childcare centres led by mothers to reduce toddler exposure to unsupervised water.
• Use of familiar community platforms, such as schools, community gatherings, and mothers' groups, to share water safety messages.
• Shared responsibility for child supervision, involving both mothers and community members, especially near high-risk water areas.

However, several recurring challenges shape the feasibility and scalability of these efforts:

• Economic constraints, including the cost of swimming lessons, transportation, and childcare.
• Inadequate local infrastructure, including the absence of swimming pools or safe, enclosed play areas, limited the feasibility of intervention delivery.
• Concerns about the safety of non-parental supervision, which may affect the uptake of formal childcare options.
• Cultural considerations, including gender norms around swimwear and instructor roles, shaped both the demand for and the design of swimming programs.
• Caregiving burdens, particularly among mothers.^16^^,^^17^

From a policy perspective, these findings highlight the need for implementation-sensitive policies that account for local context, capacity, and constraints. The findings also reaffirm that communities are not passive recipients but are willing and capable partners in drowning prevention, provided they are meaningfully engaged, equipped with essential knowledge, and supported through policies that create conditions or environments that are needed for effective implementation, as well as modest, well-targeted investments.

### Policy integration and governance gaps

With the extensive government structure in Indonesia, it is understandable that participants in this study, primarily low-income parents and village leaders with limited formal education, could not always identify specific policy actors. However, their experiences expose systemic gaps in the availability and accessibility of drowning prevention strategies, that suggest that drowning prevention has yet to become a national priority in Indonesia.

Despite a high burden and known risk due to its geography, Indonesia lacks an integrated drowning prevention framework.^6^ Although the Indonesian Ministry of Health initiated the development of a National Drowning Prevention Strategy and Coordinating Agency in 2020 in response to WHO's call and the 2021 United Nations General Assembly (UNGA) Resolution to strengthen national drowning data and governance mechanisms, its progress remains fragmented.^6^ As highlighted in our previous baseline scoping review on drowning prevention in Indonesia, including analysis of the relevant policy frameworks, this effort has yet to result in a coherent, intersectoral policy architecture.^6^ In practice, drowning prevention in Indonesia remains siloed across disparate domains, including health, education, early childhood development, transportation safety, disaster risk reduction (DRR), maritime safety enforcement, and spatial planning, with limited intersectoral collaboration or shared funding mechanisms.^6^

While this study is not intended to be nationally representative, the findings illustrate how local community perspectives, particularly from rural, resource-limited settings, can be aligned with existing national policy mandates, to offer scalable entry points for translating grassroots priorities into actionable policy pathways, as well as bridging the gap between national intent and local implementation by showing how current mandates and resources, such as school operational funds and regional development plans, can support community-led initiatives without requiring new legislation, as outlined in Table 2. However, translating policy intention into action will require a comprehensive situational assessment to map existing efforts, identify policy gaps, and evaluate financing and workforce capacity across national to village levels.

**Table 2:** Mapping community findings to relevant Indonesian policy sectors, lead agencies, and legal frameworks.

Sector/theme	Lead agencies	Relevant findings from this study	Existing policy/law (with description)	Policy gaps identified	What this study adds
Drowning prevention strategy	Ministry of Health (MoH)	Community prioritised swimming lessons, but access is limited, costly, and inconsistent in curriculum.	• MoH-led Drowning Prevention Initiative (2020): Preliminary situational assessment on drowning burden and prevention in Indonesia.^20^	No integrated, funded national drowning prevention strategy; limited implementation of MoH 2020 roadmap	Highlights strong local demand for basic swimming education and the gap in program delivery; provides community-informed, low-cost implementation routes that can operationalise WHO and United Nations General Assembly's frameworks.
Boating & maritime safety	Ministry of Transport; Indonesian Maritime Security Agency, Sea and Coast Guard; Harbourmasters	Participants reported unsafe boat travel (e.g., lack of life jackets, safety info) on inter-island routes.	• Law No. 17/2008 on Shipping: Regulates various aspects of maritime activities in Indonesia, including water transportation, port operations, and maritime safety and security.^21^ • Presidential Regulation No. 178/2014: Coordinates maritime security and rescue operations within Indonesian waters through Indonesian Maritime Security Agency.^22^ • Law No. 32/2014: Addresses marine affairs and safety in coastal and small island regions, including marine conservation, marine spatial planning marine disaster management, and safety in shipping.^23^	Poor enforcement of safety regulations; limited community awareness of maritime safety protocols	Provides grassroots data on routine unsafe boat practices and need for basic enforcement of boating safety regulations in underserved inter-island areas.
Disaster risk reduction (DRR)	National Disaster Management Agency; Meteorology, Climatology, and Geophysical Agency	Participants noted lack of flood or tsunami early warnings and absence of risk communication in villages.	• Law No. 24/2007: Concerns disaster management in Indonesia, establishes framework for disaster preparedness, including floods, and outlines the responsibilities of various government levels.^24^ • Law No. 29/2014 on Search and Rescue (SAR): Defines search-and-rescue responsibility including disaster events and emergencies, including related on operations on waters.^25^ • Law No. 17/2007 on the National Long-Term Development Plan (RPJPN) 2005–2025, including on strengthening health and education services as part of human resource development.^26^ • Law No. 59/2024 on the National Long-Term Development Plan (RPJPN) 2025–2045, including on resilience against disasters and climate change.^27^	DRR not linked to everyday drowning risks or seasonal water hazards in policy or community outreach	Highlights need to link DRR systems with local drowning risks; suggests integration of seasonal flood and water safety messaging into DRR communication.
Integrating water safety into early childhood services	Ministry of Education and Culture (MoEC); Ministry of Women Empowerment and Child Protection (MOWECP)	Participants supported community childcare to reduce child exposure to water but raised concerns over affordability, safety, and trust in non-parental supervision.	• MoEC Early Childhood Education (PAUD) Program: Focuses on early childhood care, development, and education for children aged 0–6 years, including play-based learning and school readiness.^28^ • MoEC Regulation No. 84/2008: Promotes gender-responsive education and safe learning environments.^29^	No linkage between drowning prevention and early childcare policies; low access to subsidised childcare; limited integration of caregiving and water safety training within early learning environments.	Positions community-led childcare as a drowning prevention tool; suggests linking safety infrastructure and training to early childhood service provision
Community infrastructure & barriers	Ministry of Public Works; Regional Development Planning Agency; Local Governments	Participants expressed concern about environmental risks, such as open wells, ditches, and unfenced water bodies, and proposed basic physical modifications (e.g., well covers, fencing).	• Ministry of Home Affairs Regulation No. 86/2017 on long-term, medium-term, and short-term regional development planning, includes provisions for budgeting.^30^ • Law No. 6/2014 on Village Governance: Establishes the legal framework for village governance in Indonesia, including the management of village funds (Dana Desa), which allows villages to fund locally prioritised public works and participatory initiatives, although drowning prevention and child safety protection are often not yet explicitly included.^31^	The regional development planning for West Nusa Tenggara currently lacks specific provisions for drowning prevention, current rural infrastructure guidelines do not explicitly address water safety or drowning prevention; community access to resources for environmental risk reduction is limited.	Highlights the need to incorporate drowning risk mitigation into local infrastructure planning and suggests that existing decentralised funding mechanisms, though not mentioned by participants, could potentially support simple, community-prioritised risk reduction measures if appropriately aligned and resourced.
Public awareness & education	MoH; Ministry of Communication and Information; Local Authorities	Social media seen as powerful channel; interest in first aid and water safety education	• MoH Regulation No. 585/2007 on guidelines for health promotion at the Community Health Centers (Puskesmas), mandating structured health education activities both within healthcare facilities and through outreach.^32^	No coordinated national drowning awareness campaign; limited structured health promotion around water safety in schools and communities.	Demonstrates high community receptivity to contextualised safety education integrated into existing structures and informal delivery platforms (e.g., Posyandu (monthly village-based maternal and child health posts), community gatherings, mothers' group).
Gender & cultural norms	MoEC; MOWECP	Participants noted discomfort with male instructors teaching girls, concerns over modesty and age	• MoEC Regulation No. 84/2008: Gender-Responsive Education Guidelines aim to respect cultural norms in schools; does not address water safety training.^29^	Cultural and religious considerations are limitedly integrated into program design.	Suggests embedding cultural sensitivity (e.g., female instructors, modest swimwear) into future swimming program design and training.
Funding & governance	Coordinating Ministry for Human Development and Cultural Affairs; MoEC; Ministry of Home Affairs (MoHA); Provincial and District Governments (Governors, Mayors, Regents); Provincial and District Education Offices; Village Governments (*Kepala Desa*)	Communities called for subsidies, local leadership, and inter-agency collaboration (e.g., SAR, education, health). While participants did not explicitly mention national funding instruments, their proposals suggest alignment with existing local development mandates that could be supported by provincial, district, or village budgets.	• Law No. 6/2014 on Villages (Undang–Undang Desa): Authorises Dana Desa to support community-prioritised development activities, including public health and child protection, although drowning prevention is not yet a named priority.^31^ • Presidential Decree No. 144/2024 on the Coordinating Ministry for Human Development and Cultural Affairs, mandating an integrated, cross-sectoral delivery of programs across ministries and local governments, including for health, education, and social welfare programs.^33^ • Minister of Education and Culture Regulation No. 16/2022 on Operational Assistance for Schools (BOP): Allows use of education funds for education and extracurricular activities, but water safety has yet to be formally integrated.^34^ • Regional Development Planning Frameworks: Enable district and provincial governments to initiatives through sectoral and cross-sectoral planning, providing an opportunity to incorporate drowning prevention into existing plans, although drowning prevention is not yet a named priority.^30^	Competing public priorities; fragmented planning processes between agencies and sectors; limited multisectoral budget coordination; lack of dedicated budget lines or explicit prioritisation of drowning prevention within health, education, and regional development frameworks, particularly in West Nusa Tenggara Province.	Supports the development of integrated local plans within existing governance structures; illustrates how provinces, districts, and villages could contribute to drowning prevention through context-appropriate use of regional or village funds, without requiring new legislation.

Despite these available frameworks outlined in Table 2, current implementation remains fragmented. The regulatory environment is marked by a lack of integration, overlapping mandates, and minimal cross-agency funding between ministries/agencies. These challenges mirror the lived experiences described by community participants in our study, where water safety interventions, if available at all, are often delivered in a fragmented, inequitable, and reactive manner.^6^

To move from policy intention to implementation, we recommend a national situational assessment to: 1) identify ongoing efforts and evaluate policy gaps; 2) map relevant stakeholders across levels of government and civil society; and 3) assess workforce and financing capacity at national, provincial, district, and village levels. Such an assessment would create the foundation for a cost-sensitive, integrated response tailored to Indonesia's complex geography and social landscape.

### The role of NGOs and non-state actors

Notably, participants did not mention the role of NGOs or not-for-profit groups, likely reflecting their limited presence in the study areas, and revealing how communities implicitly viewed the public sector as the sole provider of drowning prevention, underscoring their reliance on government-led delivery rather than NGO engagement. Unlike in many HICs, where NGOs and lifesaving organisations play a central role in drowning prevention, such actors are far less visible in rural Indonesia, where public sector programs lead most health and safety efforts. Furthermore, while we acknowledge the relevance of participatory coastal research across HICs,^29^, ^30^, ^31^, ^32^ the drowning prevention landscape in our study settings differs both structurally and culturally. In most of our study locations, NGOs, lifesaving initiatives, and volunteer lifeguards were largely absent. Instead, participants identified public-sector-led platforms as more realistic and trusted channels for implementing prevention efforts.

Nonetheless, we recognise that examples from other contexts can offer valuable insights around policy opportunities and actors for strengthening community engagement and capacity.^35^, ^36^, ^37^, ^38^, ^39^, ^40^, ^41^, ^42^ Non-state actors with technical expertise in drowning prevention can be key partners in designing and implementing locally adapted strategies, such as swim training, community supervision models, and low-cost safety infrastructure, such as demonstrated in Bangladesh.^33^^,^^34^^,^^43^^,^^44^ They can also support capacity building for volunteers and caregivers and help connect communities with local authorities and academic institutions.^33^^,^^34^^,^^43^ To harness these strengths, national policies should establish mechanisms for government–NGO-academic collaboration, as well as incentives for broader participation, particularly in underserved areas.^34^^,^^35^^,^^45^

### A bottom-up model: rethinking implementation pathways

Crucially, the qualitative data revealed a shared belief that drowning prevention can be anchored in existing routines and relationships, rather than imposed through top-down campaigns. Community input can be translated into practical ways to localise national policies:

• Funding: Allocate school, village, or regional development funds for safety infrastructure (e.g., well covers, ditch barriers) and swimming lessons.
• Education: Strengthen the integration and delivery of school-based swimming training.
• Infrastructure: Integrate simple, low-cost safety improvements (e.g., putting playgrounds further away from water bodies, fencing water bodies) into rural development plans.
• Peer Education: Train local volunteers (not just professionals) e.g., to deliver swimming lessons and water safety education, safe water rescue and resuscitation.
• Childcare: Align informal caregiving models with early childhood protection policies, engaging not only mothers but also community members in shared supervision.
• Gender Sensitivity: Ensure program design accommodates cultural preferences (e.g., female instructors, modest attire).
• Community Health: Use trusted community platforms, such as *Posyandu* (monthly, community-led maternal and child health posts exist in almost all Indonesian villages), school programs, and cultural gatherings, to deliver water safety education.

Although participants did not explicitly reference policy instruments or funding mechanisms, their suggestions resonate with existing policy frameworks, such as Regional Development Planning, Village Funds, and School Operational Funds. These findings suggest a potential shift from top-down campaigns toward local mobilisation of communities, provided they are informed and supported through appropriate policy alignment.^29^^,^^46^^,^^47^

**Fig. 2:**
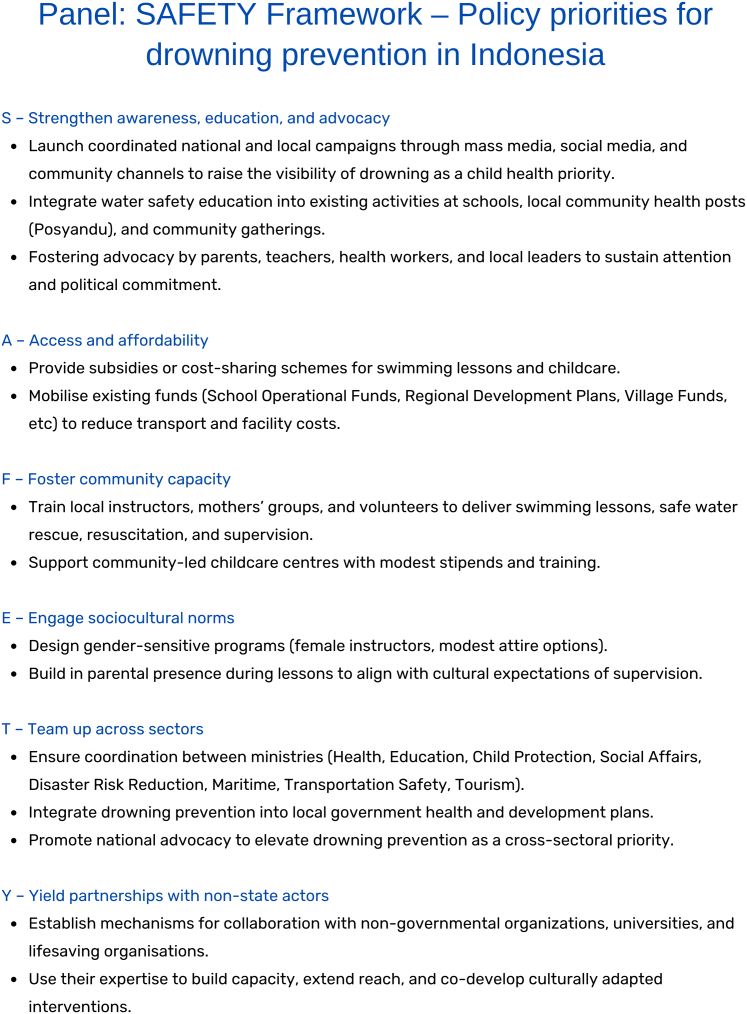
SAFETY framework—policy priorities for drowning prevention in Indonesia.

Our policy priorities can be summarised under the SAFETY framework (Strengthen awareness, education, and advocacy; Access and affordability; Foster community capacity; Engage sociocultural norms; Team up across sectors; Yield partnerships with non-state actors), outlined in the Panel (Fig. 2).

### Limitations

This study was conducted on a single island in Indonesia, which may limit the generalisability of its findings to other regions within the country and to other LMIC settings. While it offers useful context-specific insights into child drowning prevention, the findings should be interpreted with caution when considering wider applicability. The use of self-selection, purposive, and snowball sampling methods also introduces potential bias and limits the transferability of results to broader populations. These limitations underscore the need for further research that encompasses broader geographical regions and explores policy relevance and application across wider Indonesian settings and in other LMICs.

## Conclusion

This study provides practical, grounded insights into how global drowning prevention guidance can be localised and implemented through community-anchored pathways. The findings underscore the need for horizontal coordination across ministries and alignment with local governments to enable structures that support community-led implementation. However, for the communities to meaningfully engage, they must first be equipped with basic knowledge to recognise drowning risks and prevention options. The findings also highlight the need for more context-specific, policy-engaged research across Indonesia's diverse regions.

## Contributors

MC, RCF, and SGD conceptualised the study. MC, RCF, and SGD contributed to data curation, formal analysis, methodology, resources, validation, visualisation, and writing (original draft and review). RCF and SGD also provided funding acquisition and supervision. PBF contributed to data curation, investigation, and validation, and writing (review). All authors critically reviewed the manuscript, had full access to all the data in the study, and had final responsibility for the decision to submit for publication.

## Data sharing statement

The direct and anonymised quotes supporting this article are available within the article itself. All anonymised transcripts are available at the James Cook University Research Data Management Repository. Conditional access to this data is subject to ethical approval to ensure compliance with ethical approval protocols and privacy considerations for the study participants. All the authors involved in the study have access to the data collected as part of this manuscript.

## Ethical considerations

Ethical clearance was obtained from the University of Mataram, Indonesia (Ethics Approval number 044/UN18.F8/ETIK/2024) and registered by James Cook University's Human Research Ethics Committee (External HREC Approval Acknowledgement reference number H9088). Prior to the FGDs, participants provided written informed consent, including agreement to participate and be audio-recorded. Consent details covered the study's objectives, data collection methods, confidentiality, and the right to withdraw anytime without penalty. All data were kept confidential, anonymised, and analysed collectively across all FGDs to prevent identification, with only non-identifying quotes used in the article. The research team ensured privacy protection and compliance with data management protocols.

## Declaration of interests

RCF reported receiving financial support not related to this manuscript from Global Advocacy Incubator – Drowning Prevention Emerging Leaders Program, and have leadership or fiduciary role, paid or unpaid, in Royal Life Saving Society – Australia and Kidsafe Australia. All authors declare no other conflicts of interest.
