# Supplementary material for: Adapting WHO drowning prevention strategies for children in Indonesia: barriers, enablers, and policy perspectives for LMICs
Source: Lancet Reg Health Southeast Asia. 2025 Dec 2;43:100697. doi: 10.1016/j.lansea.2025.100697 (PMC12719677; doi:10.1016/j.lansea.2025.100697)
Supplement: Supplementary Table S1 [file mmc1.docx]

Supplementary Table 1. Definitions and illustrative quotes supporting interpretations of themes

| Themes | Definition and inclusions | Sub-themes | Representative excerpts |
| --- | --- | --- | --- |
| Perceptions of acceptability towards WHO-recommended interventions | Participants’ perceptions on the extent to which WHO-recommended interventions will be embraced and supported by community. | Swimming lessons for school-age children | *“It would be beneficial for the kids to have swimming lessons in this community, provided by the government, but only if we can afford them. Most importantly, the lessons need to be available locally so we don’t have to travel far*.” (Group 2, female, participant ID number: G2F4)  *“There are swimming lessons provided by the school, but only for junior high school. About twice a year. Once every semester. They call it grading, grading the swimming ability and that’s it. But it is far to swim. My kids are in junior high school, but I still need to drop my kids at the swimming pool, because they cannot ride motorbike. Even the cost of having them go for swimming [lessons arranged by the school] twice a year is already difficult for me, let alone more often than twice a year. If it’s, say, once a month, oh no. So if there is a pool around here to swim, the children would love it very much. .. As long as it has swimming instructors. We don’t have anyone that can teach them to swim around here.”* (Group 2, male, participant ID number: G2M2)  *“Many parents would be so glad for that, having their children have swimming lessons, especially if provided by the government.”* (Group 4, female, participant ID number G4F4)  *“Yes, we are very much open to the idea of swimming lessons for children in local water bodies here. If there is a facility around here provided by the government, a location to learn to swim, and it is clean, that should be enough.”* (Group 1, female, participant ID number: G1F5) |
|  |  | Supervised safe places for children | *“If there is a childcare facility in this village, to have the children being watched, that would be helpful to stop the children from playing in streams.”* (Group 6, female, participant ID number: G6F1)  *“People will welcome a childcare centre in the community to prevent drowning, especially as mothers would be busy cooking and doing other things.”* (Group 4, female, participant ID number: G4F4)  *“Such as having an early childhood education centre in the community. That would be a good idea to prevent child drowning.”* (Group 4, female, participant ID number: G4F3) |
|  |  | Training parents and community members in first aid | *“We do not know how to provide first aid for drowning. We really need that kind of education. Mothers need to be trained.”* (Group 6, female, participant ID number: G6F3)  *“I think the local government, relevant government departments should provide education for us on how to rescue children. The most important thing is education. Parents need to be informed, for those whose children are already drowned, already in the water, their lives could be saved, actually. But you need to know how to do it. So, first aid training, we need the education. We cannot perform rescue on people if we do not have the knowledge. So perhaps a training session, with mannequins. These mothers have great memories, great minds. They could learn fast. But when it comes to your own children drowning, you will panic and forget things, so you need to train them.”* (Group 6, male, participant ID number: G6M1)  *“For us, for instance, we perhaps have only watched in on tv, how to save people who are drowned. But we do not know how to do it.”* (Group 6, female, participant ID number: G6F1) |
|  |  | Installing barriers to limit access to water bodies | *“We wish for the people to have their wells covered. At least with removable lids, to cover the wells when they are not used.“* (Group 2, male, participant ID number: G2M2)  *“We used to have an open well here in the house, but someone fell into it. So, we covered the well. We covered it with bamboo, after someone tripped and fell into it.”* (Group 4, female, participant ID number: G4F5)  *“Maybe you can put fences around the streams. Perhaps not covering it but fencing it, such as with fences from bamboo. So that the kids cannot climb over it.”* (Group 3, female, participant ID number: G3F2) |
|  |  | Enforcement of boating and maritime safety regulations | *“No life jackets [on board public boats] … There were life jackets on the top deck, but we were not given them to wear. No one gave instructions.”* (Group 6, male, participant ID number: G6M1)  *“No one on board of boats uses life jackets. Never. The boat crews never let people know that we must use life jackets and where the life jackets are stored. And I have taken a few boat and ferry rides, to Bali and to Java. But there is no such information on life jackets. There should be life jackets being prepared, as it should. Sometimes there were life jackets around, sometimes no life jackets can be seen around. But no, there was no information on that. Especially on smaller boats. No one tells you what to do.”* (Group 2, male, participant ID number: G2M1)  *“No, we did not wear life jackets on boat. There were no life jackets. There was no safety equipment whatsoever.”* (Group 3, female, participant ID number: G3F4) |
|  |  | Managing risks of flood and tsunami | *“Yes, we wish to be alerted and informed about tsunami. In case of earthquakes, what we should do.”*Group 1, female, participant ID number: G1F6  *“The water level needs to be monitored, not to be over their capacity and flood.”* (Group 6, male, participant ID number: G6M1)  *“Maybe of the government, our hope is to be informed better on the threat of tsunami.”* (Group 1, female, participant ID number: G1F5) |
| Barriers of drowning intervention implementation | Participants’ perspectives on factors that may pose challenges to the effectiveness and sustainability of drowning prevention strategies to be implemented in the community. | Limited awareness on drowning risks | *“No, we don’t think about drowning. Maybe drowning happens on the coast, near the sea. We only have ditches and streams around here. We don’t really think of drowning because we already have other problems. Nothing serious, just common childhood illnesses.”* (Group 1, male, participant ID number: G1M1)  *“I don’t think people here consider drowning a concern. Many here are a bit scared of water because we don’t know how to swim.”* (Group 7, female, participant ID number: G7F2)  *“No one supervises the children while they play around the streams. They often play together with other young kids. By sunset, their mothers come searching for them. We just let them [children] be. Children look after one another. Many ditches in this village are also not covered.”* (Group 1, female, participant ID number: G1F7) |
|  |  | Financial considerations | *“Paying for it [swimming lessons] is difficult because you need to pay for everything, including transport cost, pool’s entrance fee, pocket money. And I have twins, so I need to pay around 100 thousand rupiahs [USD 6] every time they go swimming. It is difficult.”* (Group 7, female, participant ID number: G7F2)  *“Funding it [swimming lessons] is the main barrier. That is the main issue. We need to pay for their entrance fee [to swimming pools], and their pocket money.”* (Group 7, female, participant ID number: G7F1)  *“To attend the childcare centre, the kids would ask for pocket money. The kids always ask for pocket money whenever they leave home. Let us say they ask for 20 thousand rupiahs [USD 1] of pocket money. That’s a lot.”* (Group 4, female, participant ID number: G4F3)  *“If I have to pay for the caretaker's fee at the childcare centre, I would prefer not to. I would rather use that money to buy rice for my children.”* (Group 3, female, participant ID number: G3F4) |
|  |  | Distance to facility | *“If the swimming lesson is being held far away, it is almost as good as none. The transport fee will be expensive for us to afford. We cannot afford it if it is far away.”* (Group 3, female, participant ID number: G3F4)  *“The distance matters a lot. No one will be able to take the children if it is too far.”* (Group 4, female, participant ID number: G4F4)  *“The location of the swimming facility is very important. If it is far and we do not have a motorbike, then we cannot take the children.”* (Group 5, female, participant ID number: G5F1) |
|  |  | Reluctance to delegate child supervision duties | *“Even if it’s someone from our own community being recruited to care for these children at the [community] childcare centre, it’s still a concern. Delegating that responsibility and trusting others with our children is difficult.”* (Group 5, female, participant ID number: G5F6)  *“Whether insiders or outsiders [of the community] are hired to supervise the children in the community-based childcare centre, I think it will still be difficult to delegate supervision. It is not easy to trust others with the care of your children.”* (Group 5, female, participant ID number: G5F1)  *“Yes, having the children being supervised by someone else is a consideration [whether to participate or not]. That is parents’ main consideration.”* (Group 6, female, participant ID number: G6F3) |
|  |  | Doubts about community member participation | *“I do not think it [community childcare centre] will work. Mothers here have their own responsibilities at home. They cannot participate as caretakers.”* (Group 7, female, participant ID number: G7F1)  *“I think having a community-based childcare centre in the community will be too difficult. There is no one to be the caretakers. Let us say, even for myself, who am I going to watch other people’s children, when I cannot even properly watch my own children*?” (Group 6, male, participant ID number: G6M1)  *“If the caretakers in the childcare centre are recruited from mothers here, it is difficult. They have their own responsibilities and a busy schedule with their activities. Mothers have a busy day.”* (Group 6, female, participant ID number: G6F2) |
|  |  | Availability of appropriate space | *“We have no space for that many children to be kept together in one place.”* (Group 4, female, participant ID number: G4F3)  *“The space is the barrier [in realising a community-run childcare centre]. We do not have the space and place to do it.”* (Group 1, female, participant ID number: G1F5)  *“There is no space for it, the location to gather that number of children.”* (Group 6, male, participant ID number: G6M1) |
|  |  | Barrier installation practicality | *“My family shares a well with several households, and it is not covered because we still collect water from it using a pulley every day.”* (Group 4, female, participant ID number: G4F3)  *“But some wells in this community are not covered, because many people use the wells, drawing water from the wells, using a pulley. So, we cannot cover it.”* (Group 7, female, participant ID number: G7F3)  *“With ditches, for instance, you cannot really cover all of them, because of the risk of flooding. When there is flooding, if the ditches are all covered, it will be difficult for the water to flow.”* (Group 6, female, participant ID number: G6F1) |
| Sociocultural considerations of implementing interventions | Participants’ perspectives on sociocultural factors that may influence the adoption and acceptance of prevention strategies. These factors were not perceived as barriers in implementing prevention strategies. Instead, these factors informed the development of culturally sensitive interventions and were viewed as preferable by participants. | Parental presence during swimming lessons | *“I think that the parents should be present, to accompany the child during the swimming lessons. You cannot just trust the instructor with our children.”* (Group 5, female, participant ID number: G5F6)  *“Yes, we would still like to watch our own children during swimming lessons. It does not matter if they are girls or boys, kids need to be supervised from unwanted things. These days, these things could happen to both girls and boys.”* (Group 2, female, participant ID number: G2F4) |
|  |  | Gender dynamics around swimming lessons | *“I am not too comfortable with male [swimming] instructors. Just to be on the safe side. If the instructor is male, then I would like to accompany them [children] during the lessons.”* (Group 4, female, participant ID number: G4F1)  *“It’s preferable for girls to be taught by female instructors.”* (Group 1, male, participant ID number: G1M1)  *"You can't really pick between male or female swimming instructors. Most instructors will be male, and that’s not a problem because kids are still young."* (Group 4, female, participant ID number: G4F3)  *"Maybe there will be concern with teenage girls wearing swimming suits. Maybe they will prefer to wear their own clothes, to be not as tight.”* (Group 3, female, participant ID number: G3F2)  *“If the girls are older, adolescents, they may feel uncomfortable wearing swimwear. If the girls are still little, it should be all right.”* (Group 2, female, participant ID number: G2F1)  *“These days, there are modest swimsuits that will be suitable for Muslims. So, for girls to wear swimwear, it should be fine.”* (Group 2, female, participant ID number: G2F6) |
|  |  | Minimum age for swimming lessons | *“It is a no from me if the child is too young. If the child is older than 12 years old, maybe during junior high school, I will let them [swim].”* (Group 2, female, participant ID number: G2F1)  *“I will allow my children to enrol [in swimming lessons] at about the age of junior high school. Even if they are in grade sixth of primary school, I will still worry about them. So, junior high, around the age of 13 to 14 years old.”* (Group 2, female, participant ID number: G2F5)  *“I think the younger the children [enrol in swimming lessons] the better. Perhaps as young as five years old.”* (Group 4, female, participant ID number: G4F3) |
| Enablers that facilitate participation in interventions | Participants’ perspectives on factors that support the successful adoption and implementation of recommended drowning interventions in the community. | Multisectoral commitment | *“Information, education is the most important thing to be provided, by child protection agency, SAR [Search and Rescue] team, firefighters. Those involve in rescue efforts.”* (Group 6, male, participant ID number: G6F1)  *“BASARNAS [the Indonesian National Search and Rescue Agency], they are responsible [to prevent drowning]. If there are people drown on the beach, BASARNAS will search for them, right.”* (Group 3, female, participant ID number: G3F2)  *“Maybe the Department of Tourism could come here to inform us [on drowning prevention].”* (Group 7, female, participant ID number: G7F3) |
|  |  | News media’s role in enhancing awareness | *“These days, we have social media. We have seen videos of people drowning, so we became more aware of its dangers.”* (Group 5, female, participant ID number: G5F6)  *“There are more news reports on drowning these days, including from celebrities. Like, if any celebrity or their children drown, we then become more aware. The news becomes a lesson for us, that we need to be more cautious. We need to be careful, to not too easily trusting other people, including to let our children being supervised by other people.”* (Group 6, female, participant ID number: G6F1)  *“We heard information from the media, that higher locations are safer from the waves [during a tsunami event]. We also watched videos on the Aceh Tsunami, in 2004. From that moment on, we knew that following massive earthquakes, there is potential for tsunami to follow suit. So, we became more aware of that, of tsunami. … Media did not exist back then, but now we have social media, we have mobile phones to look for information on drowning.”* (Group 6, male, participant ID number: G6M1) |
|  |  | Financial subsidies | *"If the government paid for the swimming lessons, it would be very helpful. Private swimming lessons are very expensive—380 thousand rupiahs [USD 24] for four sessions. We cannot afford it."* (Group 7, female, participant ID number: G7F2)  *“If the government is willing to provide the location to swim, and have the swimming lessons for free, then it would help the community a lot. That is perhaps our suggestion for the government.”* (Group 1, male, participant ID number: G1M1)  *“Mothers who don’t work, would be willing to help in supervising children [in childcare centre], if they are being empowered financially by the government.”* (Group 1, female, participant ID number: G1F5) |
|  |  | Leveraging collectivism | *“I am willing to participate in supervising children. It is for our own community anyway. We are used to having our children being watched by others in this community. I would feel much better if there is a space for children to be supervised, so that they do not go playing too far.”* (Group 4, female, participant ID number: G4F1)  *“I think people here are used to helping each other to look after each other’s children while they have something else to do. Like your neighbours or other family members, you can ask to drop your kids at their home if you need to go somewhere. So, I think if there is a childcare centre here, for free, I think people will be willing to help each other taking care of children in the community.”* (Group 1, female, participant ID number: G1F2)  *“I think the women here would be happy to help, to participate [in supervising children in the childcare centre]. In terms of time availability, people here will be willing to participate in taking turns to supervise children in the community-based childcare centre. We are used to it.”* (Group 1, female, participant ID number: G1F5) |
